# An Unusual Case of Testicular Disorder in Sex Development of Arabian Mare (64,XX *SRY*-Negative)

**DOI:** 10.3390/ani10111963

**Published:** 2020-10-25

**Authors:** Vincenzo Peretti, Katiuska Satué, Francesca Ciotola, Santo Cristarella, Massimo De Majo, Vito Biondi, Emanuele D’Anza, Sara Albarella, Marco Quartuccio

**Affiliations:** 1 Department of Veterinary Medicine and Animal Production, University of Naples Federico II, via Delpino 1, 80137 Naples, Italy; vincenzo.peretti@unina.it (V.P.); emanuele.danza@unina.it (E.D.); sara.albarella@unina.it (S.A.); 2 Department of Animal Medicine and Surgery, Faculty of Veterinary Medicine, CEU-Cardenal Herrera University, 46115 Valencia, Spain; ksatue@uchceu.es; 3 Department of Veterinary Sciences, University of Messina, Polo Universitario Annunziata, 98168 Messina, Italy; scristarella@unime.it (S.C.); mdemajo@unime.it (M.D.M.); vbiondi@unime.it (V.B.); mquartuccio@unime.it (M.Q.)

**Keywords:** horse, megaclitoris, hypoplastic uterus, *RSPO1*, *SOX9*

## Abstract

**Simple Summary:**

An interesting case of a horse with an XX, SRY-negative disorder of sexual development (DSD) is reported in this paper. In particular, the animal showed the development of both male and female portions of reproductive organs. The possible genetic background of this abnormality is also discussed.

**Abstract:**

A 3-year-old Arabian mare underwent medical examinations due to the presence of abnormalities of the reproductive apparatus and stallion behavior (nervous temperament, aggressiveness, masculine attitude). During the clinical visit, an anovulvar distance shorter than normal was observed; moreover, vulvar lips were dorsally fused except for the lower neckline, showing a blind ending from which a penis-like structure protruded. The ultrasound examination revealed the presence of a cervix and corpus of a uterus, hypoplastic uterine horns, and small gonads with an echogenicity similar to a testis. Blood testosterone levels ranged from 0.4 to 0.6 ng/mL. Cytogenetic analysis showed a normal female karyotype (2n = 64,XX), while PCR amplification of SRY and ZFY genes revealed the absence of a Y chromosome. At necroscopic examination, internal genitalia arising from the genital ridge in the form of masculine type structures were found, while those deriving from the Mullerian ducts were of feminine type. In addition, an infundibular portion of the salpinx at the cranial pole of the gonads was found. This is the first case in equine species of DSD 2n = 64,XX *SRY*-negative, with the simultaneous presence of male (hypoplastic testicles, epididymal portions, and a penis-like structure) and female (cervix, horn and body of a hypoplastic uterus) genital structures.

## 1. Introduction

Reproduction and fertility are the most important traits in domestic animals; thus, many studies aim to identify the causes of reproduction failure in domestic species. Among the most investigated fields, there are the disorders of sex development and reproductive performances [1,2,3,4,5]. Sex chromosome abnormalities are a common karyotype aberration in horses, causing infertility or subfertility [6,7]. All congenital abnormalities in which the chromosomal, gonadal, or anatomical sex disagree are classified as disorders of sexual development (DSDs). They are often the hidden causes of economic losses in animal farming, and they have been described in all domestic animal species: buffalo [8], bovine [9], sheep [10], goat [11], pig [12], dog [13], cat [14], and ferret [15]. In horses, several types of DSDs have been reported: (i) 63,XO; (ii) 64,XX/XY; (iii) 64,XX, *SRY*-negative; (iii) 64,XY, *SRY*-positive; (iv) 64,XY, *SRY*-negative [16,17]. Molecular mechanisms underlying these conditions are only partially known. Sex-determining region Y (*SRY*) is the main gene involved in gonadal differentiation during embryonic development; its expression results in the upregulation of *SOX9* with which it provokes the differentiation of Sertoli cells and the formation of the testes. Moreover, *SRY* causes the differentiation of Leydig cells, which produce testosterone in the presence of fetal testicular androgens. The Wolffian ducts persist and develop into the epididymis, vas deferens, and seminal vesicles, and the external genital tract and prostate develop [18,19]. The characterization of the *SRY* gene in horses has allowed for the differentiation of the four possible DSD types according to the chromosomal complement detected in the individual: (1) animals with a mare-like phenotype and a male karyotype (2n = 64,XY), and (2) animals with a stallion-like phenotype and a female karyotype (2n = 64,XX), based in the presence/absence of the *SRY* gene [7,16,20]. In horses, 64,XX SRY-positive DSD cases have not been reported to date, and the most common DSD seems to be 64,XY *SRY*-negative [6], a condition probably caused by the loss of the *SRY* gene during an abnormal X-Y meiotic interchange. These animals are characterized by the presence of small ovaries, underdeveloped uterus and cervix, and external genitalia with normal appearance. On the contrary, 64 XY, SRY-positive horses show testes or gonadal digenesis [21,22,23,24,25]. In this case, SRY can be nonfunctional or other genes directly or indirectly connected to it might not be working well. In mammals, the first step for sexual differentiation is the formation of undifferentiated bipotential gonads during early embryo development. This process is under the control of different genes like *EMX2*, *LHX9*, *NR5A1*, *WT1,* and *CBX2* [26]. *WT1*, *NR5A1,* and *CBX2* are also positive regulators of *SRY* expression in the carriers of this gene, and, when levels of *SRY* reach a critical threshold, downstream genes like *SOX9* are activated so that bipotential gonads develop into testes. The overexpression of *SOX9* in XX embryonic gonads leads to the formation of testes instead of ovaries, as well as mutations in the *RSPO1* gene that alters its function [27]. 64,XX, SRY-negative males were reported in several breeds of horses [16,28,29,30,31]; their DSD is attributed to the action of an autosomal recessive gene and is therefore considered an inherited disorder [32]. The affected individuals have female sex chromosomes but show the presence of abnormal gonads, with ovarian and testicular hypoplastic tissue or ovotestis in the abdomen or in the inguinal region/canal. The other tract of genital apparatus shows alterations of various degrees that may range from mild (e.g., underdeveloped udder with a small amount of mammary tissue) to moderate but abnormal (e.g., normal vulval lips and blind-ending vaginas) [16]. The typical phenotypic aspect that may be observed in mares affected by this type of DSD is an enlargement of the clitoris that may vary from a simple hypertrophy to the appearance of a small phallus [16,22,23,24,25,26,27,28,29,30,31,32,33,34]. This report describes an unusual DSD case of a 64,XX, *SRY*-negative Arabian horse with abnormal sexual development and infertility; clinical, cytogenetic, and molecular findings are described.

## 2. Materials and Methods 

### 2.1. Ethical Statement

No ethical approval was required in compliance with European Directive 2010/63/UE and Italian Regulation D.Lgs n. 26/2014 because all the data derives from routine veterinary clinical practices.

### 2.2. Clinical Examination

A 3-year-old Arabian mare was submitted to a clinical visit at the Pathophysiology and Reproduction Clinical Unit of the Veterinary Teaching Hospital of the Department of Veterinary Sciences for the presence of ambiguous external genitalia and abnormal behavior (nervous temperament, aggression, masculine attitude).

Anamnesis reported that the mare underwent a clinical visit at the age of 18 months, at which clitoral hypertrophy and an underdeveloped internal genital system were found. In addition, the owner reported that the animal showed the typical sexual behavior of a male towards other mares in heat: sniffing, lapping of the vulvar lips, flehmen, jumping, coitals, and flag waving of the tail. During sexual behavior, the structure considered the “clitoris” stretched and enlarged so much as to resemble a small erect penis, reaching the size of about 10–12 cm in length.

**Figure 1:**
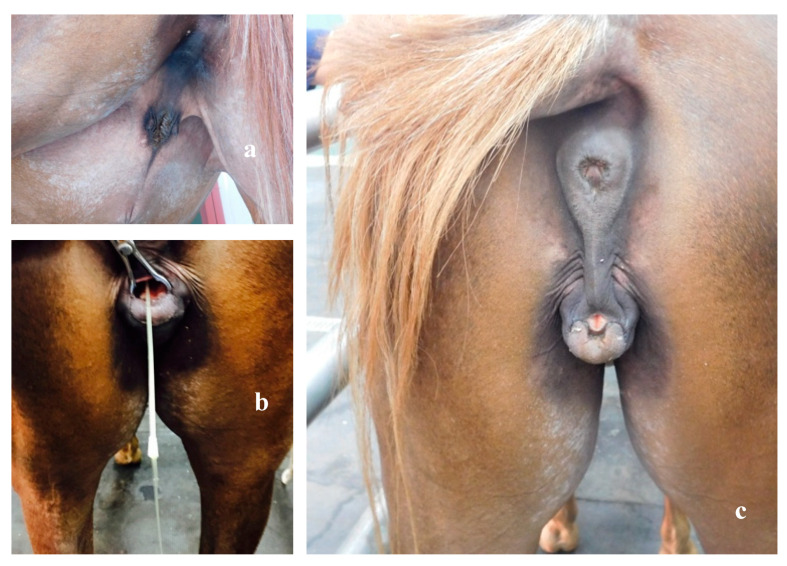
Arabian horse affected by DSD (**a**) Atropyc mammary glands. (**b**) Localization of urethral ostium and bladder; urine output through the urinary catheter. (**c**) Abnormal anus genital distance, vulvar lips fused dorsally, glans protrusion of the glans at the level of the lower vulvar commissure.

During urination, the animal assumed the typical attitude of the mare (lifting of the tail and spreading of the hind limbs). At the time of the visit, the mare was in good health and nutrition; upon inspection, it was possible to highlight the presence of the mammary glands in the groin region (Figure 1a). The animal showed an abnormal anus–genital distance, vulvar lips fused dorsally, with the exception of the lower commissure, which ended blindly, from which protruded a structure similar to a glans (Figure 1b).

After insertion of a catheter opposite the glans, the ostium and meatus urethra were located, easily passing to the urinary bladder (Figure 1c).

**Figure 2:**
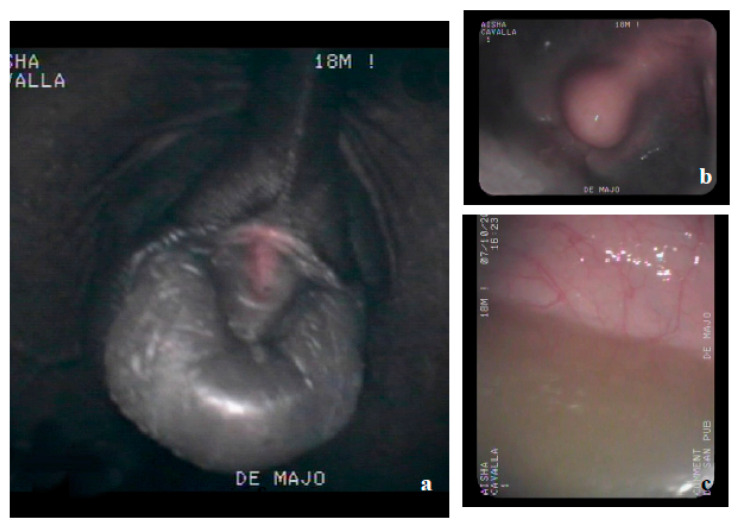
Video endoscopy of the horse. (**a**) Glans; (**b**) localization of internal urethral orifice and urethral meathus by video endoscope; (**c**) localization of bladder by video endoscope.

By means of flexible video endoscope of 10 mm in diameter, connected to a processor integrated into the Pentax insufflation system (EPK 1000 Pentax, Milan, Italy) and dorsally inserted to the glans (Figure 2a), the internal urethral orifice, urethral meathus, and bladder (Figure 2b,c) were identified. Before ending the endoscopic examination, biopsy samples were taken along the entire route. 

Immediately afterward, a rectal exploration was carried out, which caused the animal serious distress, revealing a small cord-like structure with a firm consistency, approximately 3.5 cm long, at the floor of the pelvis, attributable to the cervix. The only palpable structures had a right and left smooth surface, flaccid consistency, and were walnut-sized and lodged in the sublumbar region, caudally to the kidneys.

**Figure 3:**
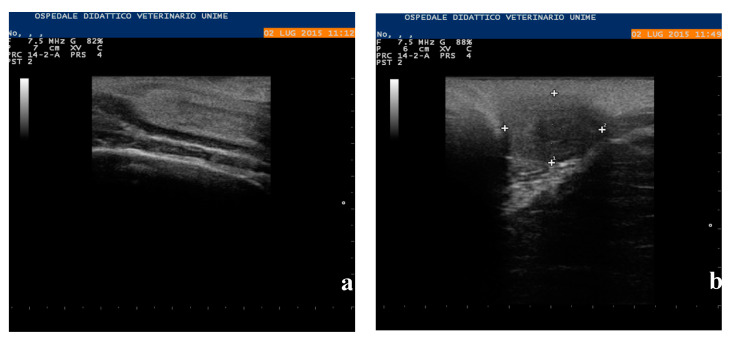
Ultrasound exam. (**a**) Cervix in **the** floor of the pelvis, with dimensions of 3.5 × 1.5 cm; (**b**) uterine horn and gonad of ovoid shape and 1.8 × 2.3 cm size.

By ultrasound examination, carried out with a portable Aquila-exhausted ultrasound system with a linear multifrequency probe (5–8 Mhz), an eco-dense structure at the floor of the pelvis, with dimensions of 3.5 × 1.5 cm, referable to the cervix (Figure 3a), was highlighted.

It was impossible to visualize the body of the uterus or the uterine horns, while the gonads were ovoid in shape and 1.8 × 2.3 cm in size; uniform and similar echogenicity to the testicular parenchyma (Figure 3b) was observed.

### 2.3. Cytogenetic Analysis

Whole blood was cultured in RPMI medium with lectin for about 72 h at 37.5 °C. Two types of cultures were set up, without and with the addition of 5-bromodeoxyuridine (BrdU; 20 μg/mL). In the latter, BrdU (20 μg/mL) and Hoechst 33258 (40 μg/mL) were added for the remaining 3.5 h. Colcemid was added 1 h before harvesting to all cultures and after a hypotonic treatment of 0.075 M KCl, and three fixations with Carnoy’s fixative cell suspensions were used to prepare slides that were allowed to dry and then stained for C- and R-banding. At least 30 to 100 metaphases were examined for karyotype analysis from slides treated for CBA and RBA-banding, respectively. Karyotype was arranged according to Iannuzzi et al. [35].

### 2.4. Hormonal Analysis

Serial bood samples were collected from the jugular vein, every 3 h for 24 h, for a total of nine samples for hormonal analysis; other blood samples were collected, always from the jugular vein, to carry out chromosomal and genetic analyses. The blood samples destined for hormonal dosages were centrifuged at 330× *g* for 10 min, and the serum was conditioned in 10 mL tubes and stored at −20 °C for subsequent analysis. The blood samples destined for chromosomical and cytogenetic analyses were collected in both 10 mL tubes without anticoagulant and lithium-heparinized tubes, placed in a container at a temperature of 5 °C, and sent to the cytogenetics laboratory of the Department of Veterinary Medicine and Animal Production of the Federico II University of Naples. The concentration of serum testosterone (T) was determined using a solid-phase I125 radioimmunoassay (Coat-A-Count Testosterone^®^, Siemens, Camberley, UK), as described by Vilar et al. [36]. The assay sensitivity was of 0.05 ng/mL, and the interassay coefficient of variation was 13.9%.

### 2.5. Molecular Analysis

DNA was extracted from whole blood using the Wizard Genomic DNA purification kit (Promega). SRY, ZFX/ZFY, and 5 exons of RSPO1 were investigated by PCR amplification (Table S1), followed by direct sequencing, according to Han et al. [37] and Ciotola et al. [33].

### 2.6. Histopathological Methods

For histopathologic examination, several samples of testes and epididymis collected during necropsies were fixed in 10% buffered formalin, included in paraffin, and sectioned at 4–5 microns thick. Subsequently, these sections were stained with hematoxylin–eosin and examined using light microscopy.

## 3. Results

### 3.1. Cytogenetic and Molecular Analyses

**Figure 4:**
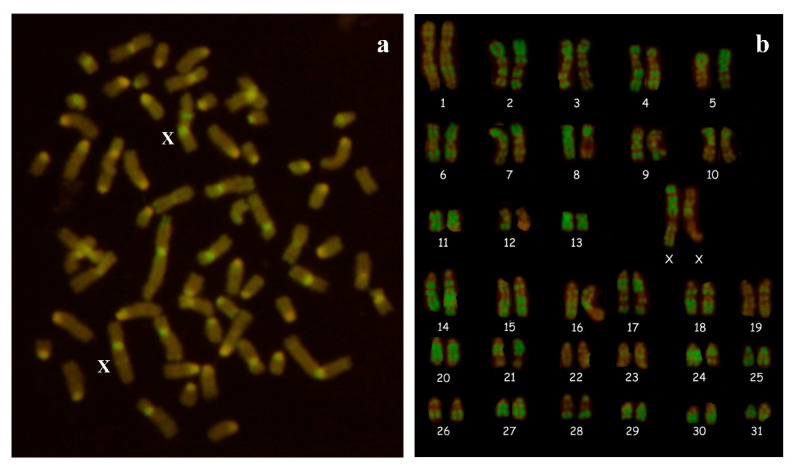
Cytogenetic analysis. (**a**) C-banded metaphase plate with 2n = 64,XX and (**b**) R-banded karyotype with 2n = 64,XX of the Arabian horse with ambiguous genitalia.

Cytogenetic analysis revealed a normal female karyotype with 2n = 64,XX (Figure 4). CBA pattern was normal in the 200 methapases examined, and no Y chromosome was identified. The absence of the Y chromosome, or part of it, was confirmed by PCR analysis. PCR amplification of the *SRY* and *ZFY* genes was negative in the blood cells of the studied intersex horse. The exons of RSPO1 showed no mutations when compared with the original sequence (NC_009145).

### 3.2. Testosterone Concentrations

The serum T concentrations ranged from 0.4 to 0.6 ng/mL. Similar T concentrations have been described in 64,XX, SRY–negative horses of this breed [33] and by Lusitano [34]. Since Kent et al. [38] reported that T concentrations varied from nondetectable to 5.4 mg/mL (stallion: 0.5–4 ng/mL; cryptorchis: 0.1–1.3 ng/mL; gelding: <0.04 ng/mL), these values indicate the presence of testicular tissue. However, in presence of hypoplastic testes, T concentrations of 0.03 ng/mL were found [30].

One month after the last visit, the animal suffered a bone fracture in the forelimb and was euthanized; hence, the anatomical and histopathological examinations were reanalyzed.

### 3.3. Necropsy and Anatomopathological Examination

**Figure 5:**
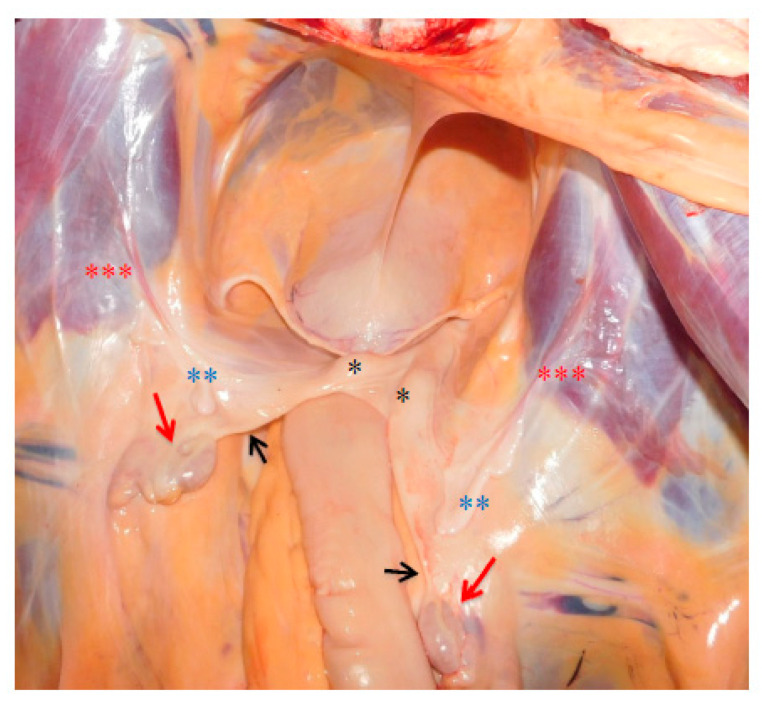
Anatomopathological examination: gonads (red arrows →) connected to the apex of the uterine horns by a cord-like structure (black arrows →). Hypoplastic horns and corpus of the uterus (black asterisk *). Two bilateral sacciform structures (blue asterisks **), located between the gonads and the uterine horns, on the lateral band of the wide ligament in caudoventral direction (red asterisks ***), up to the internal inguinal ring (a typical characteristic of the cremaster muscle).

At the internal examination of the abdominal cavity, two gonads placed at the level of the sublumbar region, posterior to the caudal poles of the kidneys, were identified. Both gonads were connected to the apex of the uterine horns by means of a cord-like structure. The horns and the corpus of the uterus were hypoplastic. Two bilateral sacciform structures, located between the gonads and the uterine horns, on the lateral band of the wide ligament, and continued in the caudoventral direction, up to the internal inguinal ring, changing color and form, assumed the typical characteristics of the cremaster muscle (Figure 5).

**Figure 6:**
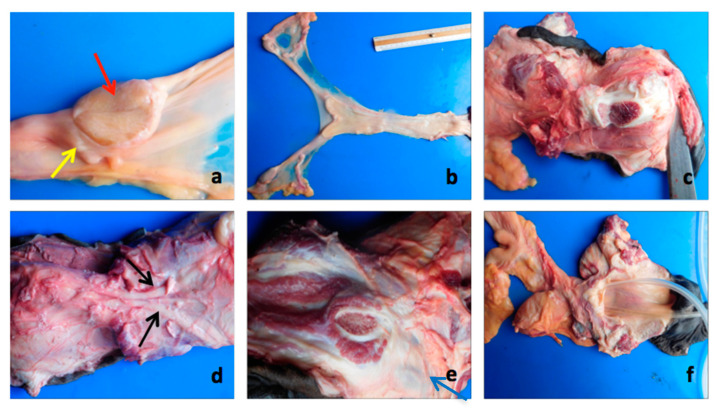
Anatomopathological examination. (**a**) Section of gonad (appearance to testicular parenchyma (red arrow →)), absence of the mediastinum and ausent follicular structure. Tubular-like structure in cranial area partially circumscribed the gonad (yellow arrow →). (**b**) Uterine horns (6 cm) and uterine body (15 cm) and cervix (3 cm). In the longitudinal section, the longitudinal folds of the endometrium are visible. (**c**) Typical structures of the corpora cavernosa. (**d**) Retractor muscles of the penis (black arrows). (**e**) Ischiocavernous muscle and bulbourethral glands (2 cm; blue arrow →). (**f**) The opening performed on fused vulvar lips led directly to the dilated pelvic urethra, helping us to locate the vaginal ostium of the cervix.

The left gonad measured 3.5 × 2 × 1 cm, while the right gonad was 3.5 × 1.5 × 1 cm; they were ovoid in shape, with a smooth surface and almost flaccid consistency. In the section, both gonads had an appearance similar to the testicular parenchyma, with the absence of the mediastinum and devoid of any follicular structure. Near the cranial pole, it was possible to highlight a tubular-like structure with a convoluted course, which partially circumscribed the gonad (Figure 6a).

The cord-like structure that connected the gonads with the apex of the uterine horns measured 14 cm on the right and 12 cm on the left.

The uterine horns measured 6 cm and were blind-bottomed. The body of the uterus measured 15 cm, and, finally, the cervix measured 3 cm. In the longitudinal section, it was possible to highlight the typical longitudinal folds of the mare endometrium (Figure 6b).

The external genital tract consisted of a very small vulva. The dorsal portion of the vulvar lips was completely fused, and the lower commissure was completely occupied by a structure comparable to the glans of a stallion.

The anatomopathological examination revealed that the glans was part of a real small penis; this was confirmed by the presence of the typical structures of the corpora cavernosa (Figure 6c), retractor muscles of the penis (Figure 6d), the ischiocavernosus muscle, and two oval structures symmetrically arranged on the right and left (sized 2 cm), probably referable to the bulbourethral glands (Figure 6e). No other glands, such as prostate and seminal vesicles, were detected.

The opening of the fused vulvar lips led directly to the pelvic urethra; this portion turned out to be quite dilated in correspondence, of which it was possible to highlight the vaginal ostium of the cervix (Figure 6f).

**Figure 7:**
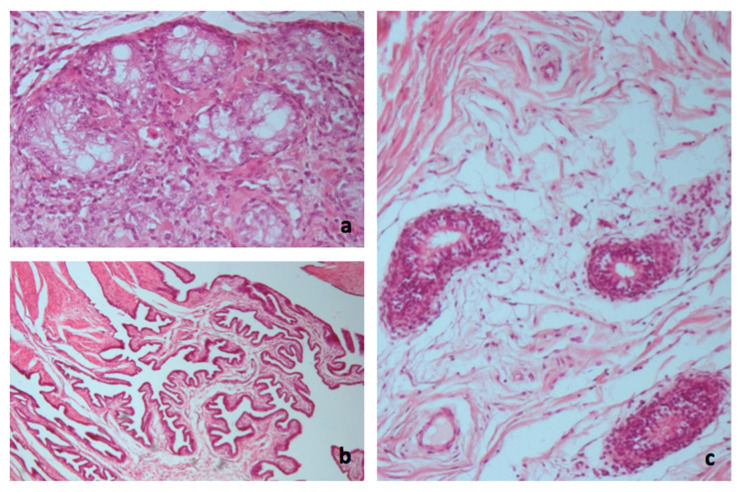
Histological examination. (**a**) Hypoplastic or atrophic seminiferous tubules, with thickening and hyalinization of the basement membrane and the presence of Sertoli cells. Complete absence of germinal epithelium. Leydig cells in the interstice with acidophilic cytoplasm (hematoxylon–eosin; ×250). (**b**) Infundibular portion of the salpinge at the level of the cranial pole of the gonads (hematoxylin–eosin; ×250). (**c**) Tubular structures covered with ciliated epithelium, referable to the epididymis or sketches of epoophoron or paroophoron (epididymis; hematoxylin–eosin; ×250).

Histological examination of the gonads revealed the presence of testicular tissue only. The seminiferous tubules were hypoplastic or atrophic, with thickening and hyalinization of the basement membrane, the presence of Sertoli cells, and the complete absence of germinal epithelium. In the interstice, there were Leydig cells with acidophilic cytoplasm. Moreover, neither ovarian follicles nor primary oocytes were identified (Figure 7a). 

The histological finding of the infundibular portion of the salpinge at the level of the cranial pole of the gonads was particularly interesting (Figure 7b). Additionally, adjacent to it, always near the cranial pole of the gonads, tubular structures covered with ciliated epithelium were found, referable to the epididymis or epoophoron and paroophoron (Figure 7c) that are a homolog to the epididymis and a remnant of the lower part of the mesonephros in the female, corresponding to the paradidymis of the male, respectively. The structure of the small penis was characterized by the presence of the corpora cavernosa, the ischiocavernosus muscle, the retractor muscles of the penis, and the presence of the bulbourethral glands.

## 4. Discussion

On the basis of cytogenetic (female karyotype) and molecular findings (SRY absence) and the presence of intra-abdominal testes and ambiguous external genitalia, the Arabian mare was diagnosed with testicular 64,XX, SRY-negative DSD or as an XX male.

Although the true cause of this disorder was not yet clarified, it is proposed that XX SRY-negative testicular DSD has, in some instances, familiar patterns of inheritance (Buoen et al., 2000), particularly in certain Arabian sire lines [38].

These types of malformations have been reported in horses of this same breed [16,33] and other equine breeds such as Pasa Fino [39], Pony [30], American Saddlebred [40], Konik [29], Andalusian [31], Tenessee Walking, Thoroughbred, Belgian [16], Lusitano [34], and others [32]. Such individuals were found XX males or XX true hermaphrodites and were infertile. 

In this case, the simultaneous presence of male gonads (hypoplastic testicles and portions of the epididymis) and female reproductive organs (horns and hypoplastic body uterus and cervix) was found. Similar evidence of abdominal testes has been found in Thoroughbred [25], American Saddlebred [40], Konik [29], Lusitano [34] and Pony [30], although they differ from Arabian and Andalusian horses, in which ovotestes were identified [16,33]. Moreover, the presence of infundibular portions of the salpynx, adjacent to the testes, was particularly interesting since it was not previously identified in the equidae with this type of genetic alteration. 

The genetic mechanism by which testes development occurs in the absence of the *SRY* gene is unclear. Other genes involved in male gonadal differentiation than *SRY* might be responsible, but they were not detected up to now [19,30,32]. *SOX9* duplications have been related to XX SRY-negative DSD in humans [41] and dogs [42]. The genomic domain regulating *SOX9* expression spans more than 1 Mb upstream of *SOX9* [43]. Benko et al. [44] revealed that a noncoding regulatory region, located far upstream of the *SOX9* promoter, was critical for gonadal *SOX9* expression and subsequent normal sexual development, and copy number variations were the genetic basis of isolated 46,XX testicular abnormality of variable severity. Moreover, the minimum critical region associated with gonadal development, approximately a 67-kb region located 584–517 kb upstream of *SOX9*, was confirmed to induce *SOX9* overexpression in female-to-male sex reversal [43]. Regulatory elements in the duplication region might enhance *SOX9* expression through complex mechanisms [45]. It was suggested that changes in *SOX9* expression, resulting from disruptions in some regions upstream of *SOX9,* could be important in the 46,XX testicular disorder of sexual development. 

The *DAX1* gene might also function downstream of the *SRY* gene in the sex-determination pathway. Overexpression of the *DAX1* gene could cause female-to-male DSD [46]. *ROCK1* (Rho-associated, coiled-coil protein kinase 1) phosphorylates and activates SOX9 in Sertoli cells to initiate testes formation [47]. *SOX3* was recently shown to upregulate *SOX9* expression via a similar mechanism to *SRY* and modulate XX male DSD in humans through gain-of-function mutations mediated by genomic rearrangements around *SOX3*, possibly leading to its altered regulation [48]. Overexpression of the *SOX10* gene at 22q13 might be another cause of testicular DSD XX SRY-negative [49]. Additionally, the deletion of the *WNT4* gene leads to the masculinization of murine embryos [50]. Finally, although the mutation of the *RSPO1* gene leads to complete female-to-male SRS in humans [27], in this case, mutations in the coding region of this gene were excluded, as previously documented in this same breed [33]. 

## 5. Conclusions

This report describes an unusual case of testicular DSD in a XX SRY-negative Arabian horse in which, for the first time, the presence of infundibular portions of the salpinx adjacent to the testes was found. The observed abnormalities are probably due to mutations in genes involved in the embryo differentiation of gonads. To analyze the sequences of all the genes that may cause the observed malformations would involve a considerable expenditure of resources. For this reason, the future perspective for this case is to analyze it through arrayCGH or SNP-chip, together with other cases of a similar phenotype, thus identifying the genetic regions and the genes to be analyzed.

## Supplementary Materials

The following are available online at https://www.mdpi.com/2076-2615/10/11/1963/s1, Table S1: Primer sequences, annealing temperatures and product lengths of SRY, ZF9 and RSPO1 gene region analysed.
