# Supplementary material for: An Unusual Case of Testicular Disorder in Sex Development of Arabian Mare (64,XX SRY-Negative)
Source: Animals (Basel). 2020 Oct 25;10(11):1963. doi: 10.3390/ani10111963 (PMC7693820; doi:10.3390/ani10111963)
Supplement: Supplementary file 1 [file animals-10-01963-s001.pdf]

Supplementary Table S1

Table S1: Primer sequences, annealing temperatures and product lengths of *SRY*, *ZF9* and *RSPO1* gene region analysed.

Gene	Primer name	Primer	annealing	length
SRY[a]	SRY-F	TGC TAT GTC CAG AGT ATC CAA CA	58	697bp
	SRY-R	TGA GAA AGT CCG GAG GGT AA		
ZF9[a]	ZF9-F	AAA TCA AAA CCT TCA TGC CAA T	58	Y 553bp; X 604bp
	ZF9-R	TTC CGG TTT TCA ATT CCA TC		
RSPO1[b]	RSPO1ex1F	CTT CCT TAT GGG CTG ATG GA	60,6	387bp
	RSPO1ex1R	AAT CTG CAA TGG TCA CCA CA		
	RSPO1ex2F	CTG CCT CTC ACT CCT GTT CC	60,6	414bp
	RSPO1ex2R	CAC ACG GAG TTG GAA ATG TG		
	RSPO1ex3-4F[c]	TCC TGT GAA ATC GGC TCT CT	60	1097bp
	RSPO1ex3-4R[c]	ACA CAG AGT TCT GGG GGT TG		
	RSPO1ex5F	GAG GGG ACC CTG ACT GTG TA	60	419bp
	RSPO1ex5R	AGA GTG TGT GTC CGT GTT GC		

[a] Han et al. 2010; [b] Gene ID: 100054745; [c] Exone 3 and exone 4 were analyzed together.
